# Functional Recovery in a Patient of Abnormal Left Parieto-Occipital Encephalomalacia With Gliosis-Associated Genu Varum Deformity: A Case Report

**DOI:** 10.7759/cureus.55115

**Published:** 2024-02-28

**Authors:** Sejal Gandhi, Anam R Sasun, Deepali S Patil

**Affiliations:** 1 Department of Neuro-Physiotherapy, Ravi Nair Physiotherapy College, Datta Meghe Institute of Higher Education and Research, Wardha, IND; 2 Department of Musculoskeletal Physiotherapy, Ravi Nair Physiotherapy College, Datta Meghe Institute of Higher Education and Research, Wardha, IND

**Keywords:** rehabilitation, case report, physiotherapy, telerehabilitation, genu varum, hemorrhage, parenchyma, hemiparesis, gliosis, encephalomalacia

## Abstract

Parieto-occipital encephalomalacia is a macroscopic appearance of the brain with loss of cerebral parenchyma associated with gliosis in the brain's anatomical structures. It occurs because of the liquefaction of brain parenchymal necrosis after cerebral ischemia, infection, and haemorrhages. It is often surrounded by glial cell proliferation in response to damage. Rehabilitation after the manifestation of neurological function must be tailored, and well-coordinated intervention must be formulated. We present a case study of a 77-year-old male with parieto-occipital encephalomalacia associated with genu varum deformity with a complaint of generalized weakness, vertigo, giddiness, and fall with one episode of a seizure attack. Further, bilateral genu varum deformity was noted on the knees. Encephalomalcia is associated with vitamin D deficiency. The physiotherapy rehabilitation consisted of resolving the symptoms of the patient, along with working on strengthening weak muscles of the genu varum deformity of the patient. The proprioceptive neuromuscular facilitation (PNF) method is a popular rehabilitation strategy for regaining motor function. Numerous outcome measures were used to monitor the patient's progress. Outcome measures such as the tone grading scale (TGS), motor assessment scale (MAS), dynamic gait index (DGI), Barthel index (BI), and world health-related quality-of-life (WHORQOL) scales were used. The rehabilitation lasted for six weeks. Tele-rehabilitation also plays a crucial impact in the recovery of patients. By the end of our rehabilitation, the patient significantly improved in performing activities of daily living and improved his quality of life. Tele-rehabilitation helped us stay connected with the patient.

## Introduction

Encephalomalacia is a rare case presentation. The brain is the most vital and complex organ, and encephalomalacia translates to "softening of the brain." Brain tissues become soft due to inflammation or haemorrhages caused by cerebral infarction, cerebral ischemia, infection, craniocerebral trauma, or other injuries known as encephalomalacia [1]. It is extremely difficult to pinpoint the precise or even estimated prevalence of osteomalacia due to vitamin D insufficiency worldwide because the ailment is frequently asymptomatic, particularly in older adults, or is frequently underdiagnosed. This disease leads to a complete stoppage of the working of the brain region involved. Encephalomalacia is one kind of infection-related brain injury. It is a chronic condition after a brain injury and is categorized as a traumatic brain injury by imaging. It affects individuals regardless of their age group. Any region of cerebral parenchymal loss with adjacent gliosis is referred to as encephalomalacia [2].

The ultimate result of insult-induced brain parenchymal liquefactive necrosis is a term that pathologists first used to explain the brain's macroscopic appearance after various traumas. Rarely have cases of patients developing left parietooccipital lobe encephalomalacia. Gliosis, or the development of glial cells in response to injury, is common around it. The symptoms and prognosis of encephalomalacia depend on the size, location, and number of lesions, as well as the presence of additional conditions, such as convulsions, hydrocephalus, or infection [3]. Although there are very few published studies on encephalomalacia in humans, the majority of the publications discuss newborns and children, very rarely adults [4]. Stroke may have contributed to the problem with blood flow. Alternative explanations include severe brain edema, which restricts cerebral blood flow. In imaging examinations, the gliosis is usually seen as a scar near the location of the brain injury. When this scar tissue in the brain contracts, it causes encephalomalacia, which can cause a variety of motor and sensory deficits [5,6].

Varum deformity is an inward angulation of the distal portion of a leg (medial angulation toward the body’s midline), also known as genu varus or bow-legged. The line of gravity passing through the lower limb joints can be disturbed by knee abnormalities, such as genu varum, which will affect both the indexes of dynamic and static balance, and this is the goal of the study [7]. The cause will dictate how a varus knee is treated. If a child has rickets, they may only need to take vitamin D or calcium supplements while the condition is still minor. In some circumstances, supplements may be sufficient to strengthen bones and alleviate problems. In moderate situations, physical therapy and weight training might help strengthen the muscles that surround your leg bones [8]. Exercise for therapeutic purposes, mobilizations, and manipulations are examples of manual treatment procedures. Prescription and use of orthotic or prosthetic devices, mobility aids, and wheelchairs; techniques for clearing airways; self-care and home care functional training; work, education, recreation, and leisure activities; and community reintegration all require functional training. Physical agents, as well as additional modalities such as hydrotherapy, electrotherapy, and cryotherapy, are used [9].

## Case presentation

Patient information

A 77-year-old male was referred from the Neurology Outpatient Department (OPD) with a complaint of right upper and lower limb weakness and clumsy balance while sitting and standing. The fault was sudden and gradually progressive. He also complained of giddiness and tingling sensations in the right half of the body. Medical history revealed the presence of acute infarctions of the left middle cerebral artery (MCA) and the posterior cerebral artery (PCA). The patient also gave a history of two episodes of seizures. Further, he revealed that he had a history of falls 15 days ago in his washroom. The patient has been a chronic alcohol consumer for 20 years. A bilateral genu varum deformity was seen.

Clinical findings

After gaining verbal consent, the examination was done. On observation, bilateral genu varum deformity was present, along with a mild deviation of the angle of the mouth. Diffuse swelling was present around the bilateral knee joint. The skin appeared tense and shiny. Tightness of the tendon Achilles and hamstring was present. On examination, speech showed dysarthria. The right upper limb and lower limb were flaccid. Tone abnormality examination revealed a +1 on the tone grading scale (Table 1). Reflex examination revealed diminished reflexes (Table 2). Balance and coordination assessments of the lower limb revealed moderate impairment, which means movements were slow, awkward, and unsteady.

**Table 1:** Tone abnormality on day one of the assessment TGS: Tone Grading Scale

Muscles	Right	Left
Shoulder flexors	+1	+2
Shoulder abductors	+1	+2
Elbow flexors	+1	+2
Wrist flexors	+1	+2
Hip flexors	+1	+2
Hip abductors	+1	+2
Knee flexors	+1	+2
Ankle plantarflexors	+1	+2
Ankle dorsiflexors	+1	+2

**Table 2:** Reflex examination on day one of the assessment

Types of reflexes	Right	Left
Superficial reflexes		
Corneal	1+	1+
Abdominal	1+	1+
Plantar	1+	1+
Deep reflexes		
Biceps	1+	1+
Triceps	1+	1+
Supinator	1+	1+
Knee	1+	1+
Ankle	1+	1+

Clinical diagnosis

**Figure 1:**
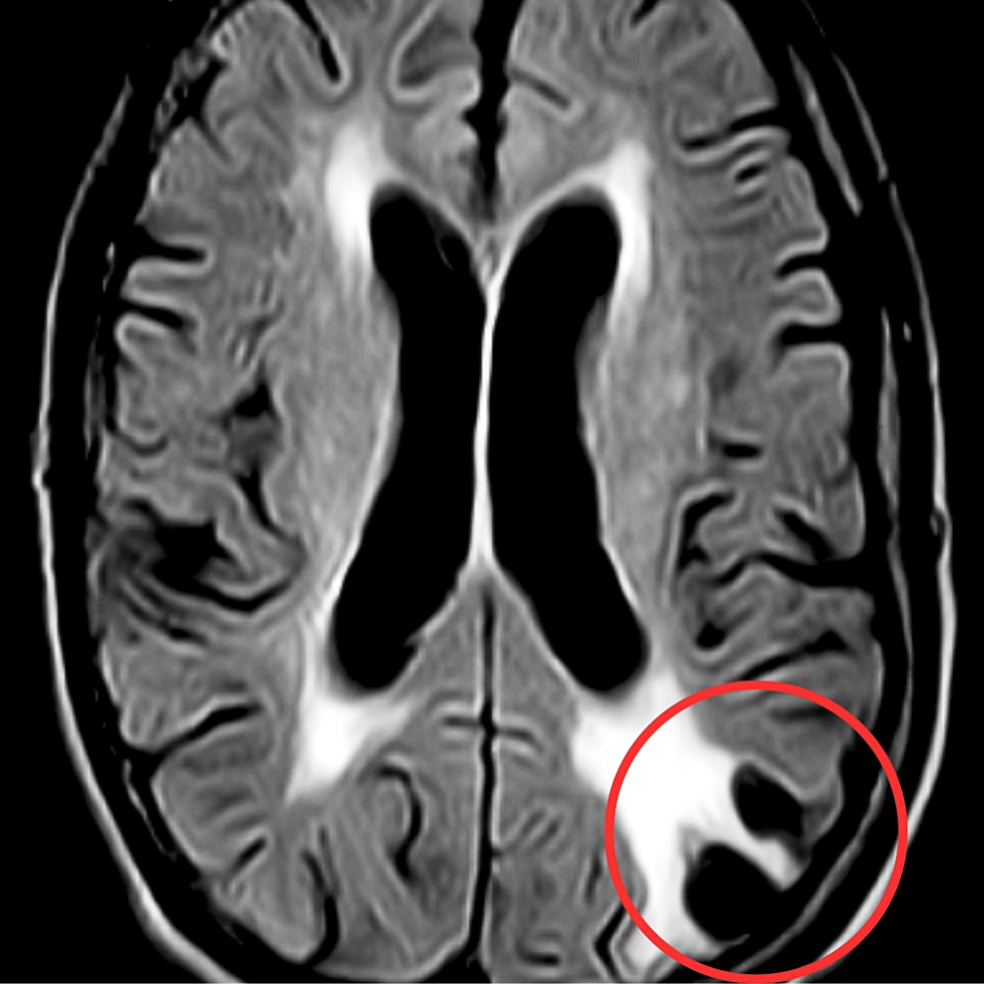
Magnetic resonance imaging of the brain The red circle shows encephalomalacia with surrounding gliosis noted in the left parietal-occipital lobe and ex vacuo dilatation of the occipital horn of the left lateral ventricle.

Magnetic resonance imaging of the brain indicated encephalomalacia alterations with gliosis in the left parieto-occipital area, as well as ex-vacuo dilatation of the occipital horn of the left lateral ventricle, which is visible in Figure 1, with prominence of sulcogyral spaces, cerebellar folia, and atrophic changes with small vessel ischemic disease.

Physiotherapy intervention

Table 3 describes the physiotherapy treatment received by the patient. Table 4 describes the outcome measures of physiotherapy rehabilitation.

**Table 3:** Pre-physiotherapy and post-physiotherapy reflexes

Types of reflexes	Pre-physiotherapy	Post-physiotherapy
Superficial reflexes		
Corneal	1+	2+
Abdominal	1+	2+
Plantar	1+	2+
Deep reflexes		
Biceps	1+	2+
Triceps	1+	2+
Supinator	1+	2+
Knee	1+	2+
Ankle	1+	2+

**Table 4:** Pre-rehabilitation and post-rehabilitation outcome measures

Outcome measures	Pre-rehabilitation	Post-rehabilitation
Tone grading system	+1	+2
Coordination examination	Grade 2	Grade 3
Motor assessment scale	30/54	50/54
Barthel index	60/100	90/100
Berg balance test	30/56	38/56
World-health-related quality of life	40/100	80/100

## Discussion

This clinical report details the physiotherapy assessment and rehabilitation of a 77-year-old male with encephalomalacia. This rare condition includes gliosis, genu varum deformity, and acute infarct in the MCA and PCA. When clinical symptoms and investigations are linked to other common encephalomalacia causes, they should be addressed immediately. Quick diagnosis and aggressive treatment yield beneficial effects.

In physiotherapy programs for old-age encephalomalacia, there is no official guideline, but the treatment of physiotherapy can help with several physical problems that encephalomalacia patients bring on. Physiotherapy aids the patient's swift return to daily activities. Regaining and relearning life skills is beneficial. The first step in physiotherapy is to evaluate and assess the medical history, range of motion, strength, neurological involvement, and functional level of the patient. After that, it creates a treatment plan based on the requirements of the patient, several other therapeutic modalities were intended, such as Rood's method, which involved applying ice (cryotherapy) to spastic muscles. This method was found to reduce spastic muscles' resistance to rapid stretching clinically and to lessen or suppress clonus [9,10].

According to Mishra et al., using electrical muscular stimulation, the weak muscle was activated or strengthened, and the muscle's performance was increased. The process of relearning motor skills after central nervous system injury [11]. According to Harris et al., the sound extremities received strength training, while the hemiplegic limbs eventually began receiving progressive resistance training. In addition to a range of motion exercise, the upper limb strength is improved via strength training. Power and function in persons with acute infarction can be improved with increasing tone and targeted rehabilitation interventions, focusing on gentle exercises, mobility training, and functional tasks tailored to individual needs and abilities [12]. Rehabilitation is required to ensure the patient's safety, freedom, and efficient movement. Successful therapy approaches improve patient outcomes by reducing issues [13].

Spinal deformity may develop from genu varum malalignment of the lower extremities, which can lead to instability of the lower extremities. Joint deterioration and abnormal gait and patellar posture are related to knee disease, and medial patellar displacement can result in osteoarthritis and knee pain [14]. In this study, a patient with genu varum has his post-exercise pain levels analyzed. The results demonstrate a substantial difference in pain levels between the groups, which is in line with the findings of the study, which found that quadriceps and neuromuscular strengthening activities reduced knee discomfort [15]. Numerous studies have shown that exercise interventions, such as quadriceps strengthening, can help individuals with knee discomfort feel better and function better. However, there were no apparent significant decreases in the varus malalignment groups. One of the characteristics of an ischemic stroke is caused by a disruption in cerebral blood flow. This decrease in cerebral blood flow causes subsequent biochemical disorders and energy failure, which in turn trigger a strong in situ inflammatory response. A complex web of connections plays a dynamic role in post-ischemic inflammation between different inflammatory chemicals and cells. The microglia, indigenous inflammatory brain cells, are especially activated in response to ischemia insults, many of which nuclear transcription factors control. Ischemic stroke is defined by the interruption of cerebral circulation.

This decrease in brain haemoglobin flow leads to additional biochemical disruptions and energy failure, which trigger strong in situ inflammatory responses. According to Davies et al., a complex web of connections plays a dynamic role in post-ischemic inflammation between different inflammatory chemicals and cells [16]. According to De Caterina et al., numerous studies conducted in the past few years have concentrated on the inflammatory responses in the ischemic penumbra zone. The inflammation developed as a secondary phenomenon in response to the acute brain tissue damage [17]. Endothelial cells that line the local cerebral blood arteries are triggered to release adhesion molecules that are responsible for peripheral circulating leukocytes to migrate into the damaged brain tissue, which promotes inflammatory signaling cascades [18]. The blood-brain barrier is damaged, which causes vasogenic edema, leukocyte secondary brain injury, and invasion. Damage to the blood-brain fence can aggravate brain tissue damage. In addition to contributing to eventual ischemia, brain injury caused by allowing blood components to reach the brain can result in further neuronal damage and exacerbate the severity of the condition [19]. The metaphyseal-diaphyseal angle of the proximal tibia is a crucial metric for distinguishing natural bowing from Blount disease, as it provides quantitative evidence of angular deformity and aids in the accurate diagnosis and appropriate management of orthopedic conditions affecting the lower limbs [20].

## Conclusions

During their physiotherapy treatment, patients with encephalomalacia with gliosis may have challenges with fatigue, weakness, pain and discomfort, decreased endurance, delayed recovery, and limited mobility. Physiotherapists and patients should work together to develop customized treatment plans that include each person's needs and limitations in light of these challenges. Among the many ways that physiotherapy can benefit individuals with encephalomalacia and gliosis are through improving physical outcomes, encouraging recovery, and lowering complications. Physiotherapy has been shown to improve physical products, prevent complications, promote rehabilitation, and improve patients' quality of life in cases of encephalomalacia. A very good form of treatment for those with genu varum is physiotherapy. Together, the patient and a physiotherapist will create a customized treatment plan that emphasizes stretching out tight muscles and strengthening weak ones. Extending tense muscles and enhancing stability and alignment all around are essential components of a comprehensive rehabilitation program aimed at improving mobility, function, and overall well-being.
